# Resveratrol Effects on Male Infertility

**DOI:** 10.31661/gmj.v13i.3622

**Published:** 2024-10-07

**Authors:** Sina Vakili, Amirabbas Rostami, Sanaz Rastegar

**Affiliations:** ^1^ Infertility Research Centre, Shiraz University of Medical Sciences, Shiraz, Iran; ^2^ Department of Internal Medicine, Faculty of General Medicine, Yerevan State Medical University after Mkhitar Heratsi, Yerevan, Armenia; ^3^ Medical Mycology and Bacteriology Research Center, Kerman University of Medical Sciences, Kerman, Iran; ^4^ Department of Medical Microbiology (Bacteriology and Virology), Afzalipour School of Medicine, Kerman, Iran

**Keywords:** Resveratrol, Male Infertility, Oxidative Stress, Inflammation, Sperm

## Abstract

The escalating prevalence of male reproductive disorders and associated infertility has become a pressing concern, necessitating heightened awareness due to its substantial socioeconomic and psychological impact. Research has elucidated the multifaceted etiology of male infertility, encompassing oxidative stress, inflammation, and lifestyle-related factors. Resveratrol, a naturally occurring non-flavonoid polyphenol, has garnered significant attention for its potential health benefits, particularly in mitigating oxidative stress and inflammation. However, the relationship between this compound and male fertility remains poorly understood, with existing research yielding conflicting results. The current review aimed to discuss the effects of resveratrol on human male fertility and explore the underlying mechanisms by which it may influence male infertility.

## Introduction

Infertility is seen as a unique medical challenge involving two individuals, leading to significant emotional and societal strain along with a substantial financial impact on patients and the healthcare sector. Male infertility is believed to impact around 7% of men globally and is a contributing factor in half of all infertility instances. Notably, research from a Global Burden of Disease study reveals that the standardized prevalence of male infertility rose by 0.291% each year from 1990 to 2017 [1]. While factors like genetics, lifestyle choices, and environmental toxins have been identified as contributors to male infertility, diagnosing the condition remains challenging with about 40% of cases falling under the category of idiopathic. Despite advancements in assisted reproductive technology offering some hope, the treatment of male infertility is still far from ideal. Efforts are underway to enhance therapeutic approaches for male infertility, focusing on the underlying factors of the disease. The pathophysiology of male infertility involves a complex network of interconnected biological pathways. Oxidative stress is a key player in the decline of male fertility indicators and a significant factor in many cases of unexplained male infertility, making the use of antioxidants crucial in treatment. However, the prescription of antioxidants comes with certain assumptions that must be reviewed carefully to avoid potential adverse effects. Numerous phytochemicals found in plants have drawn interest due to potential health benefits in treating a variety of chronic diseases. These substances have properties that may provide protection against inflammation, oxidative stress, and the development of certain diseases. Many phytochemicals are rich sources of antioxidants and have been studied extensively for their potential to treat chronic diseases associated with oxidative stress.

## 1. Search Strategy

A thorough investigation was carried out, encompassing studies published up to September 2024, without any initial exclusions. The search involved pertinent keywords such as "resveratrol", "spermatogenesis", "reproduction", "fertility", "male", "infertility", "semen", "sperm", "FSH", "LH", and "testosterone" across Scopus, PubMed, and Google Scholar search engines. Following this, studies failing to meet specific criteria were omitted from the review. These criteria encompassed non-English studies, abstracts lacking relevant details, non-original research, or studies with inaccessible findings. The studies that fulfilled the criteria were included in the review.

## 2. Pathophysiology of Male Infertility

Male infertility is a complex and multifaceted condition, involving abnormalities in semen analysis, varicocele conditions, urogenital infections, and sexually transmitted diseases, even in cases where conventional semen analysis appears normal. It can be broadly classified into four main types: central nervous system/hormonal imbalance, post-testicular, testicular, and pre-testicular causes. Central nervous system and hormonal imbalances can disrupt the pituitary gland or hypothalamus function, significantly impacting fertility.Post-testicular factors can be linked to injuries in the seminal tract, inflammatory diseases, or complications from bladder neck surgery. Testicular issues may arise from varicocele formation, epididymal dysfunction, or testicular tumors. Pre-testicular factors encompass conditions like hypogonadotropic hypogonadism, erectile dysfunction, and genetic abnormalities.

Male infertility may result from a mix of lifestyle and physiological variables. Fertility can be hampered by erectile dysfunction or early ejaculation, and sperm production may be impacted by some drugs, such as steroids and some antidepressants. Decreased sperm quality can also result from aging. Male fertility is greatly influenced by diet and nutrition, with infertility possibly being the result of poor dietary choices and nutritional inadequacies. Sperm production and function can be affected by conditions such as varicocele, infections, hormone imbalances, and genetic problems. Lifestyle decisions that negatively impact sperm quality and fertility include smoking, obesity, drug addiction, and excessive alcohol consumption. The health of sperm is at risk from exposure to radiation, environmental pollutants, and industrial poisons. Elevated testicular temperature from sources including hot tubs, saunas, or tight clothes might influence sperm production. Hormone levels and sperm production might be impacted by prolonged stress.

The most important causes of male infertility are oxidative stress and inflammation. Oxidative stress and inflammation collaborate synergistically to compromise male fertility through several pathways including mitochondrial dysregulation, diminished testosterone synthesis, and alterations in seminal fluid composition. Collectively, these factors inflict damage upon sperm cells, disrupt fundamental reproductive processes, and ultimately diminish overall fertility potential [2][3]. Hence, addressing both oxidative stress and inflammation is crucial for improving male fertility outcomes. Lifestyle modifications and potential treatments targeting these issues may help optimize reproductive health and fertility.

## 3. Resveratrol

Resveratrol (RSV), a multifaceted polyphenolic compound, boasts an impressive array of health-promoting attributes. This bioactive molecule, prevalent in grapes, berries, and various plant species, demonstrates remarkable versatility in its physiological impacts. It confers cardiovascular protection, exhibits potent anticancer properties, and neuroprotection as well as displays anti-inflammatory characteristics, and may potentially mitigate symptoms of aging. The dietary consumption of RSV is notably limited, approximating merely 100 micrograms per day. This compound exhibits excellent bioavailability, undergoes rapid metabolism, and is primarily excreted via urinary elimination. The distinctive molecular architecture of RSV underpins its broad spectrum of biological activities, rendering it an intriguing subject for ongoing scientific investigation and potential therapeutic exploration [4][5][6].

## 4. RSV Effects on Male Fertility

**Table:** Table1. Overview of Main Research on Resveratrol’s Impact on Male Fertility

**Study**	**Specie**	**Resveratrol Dose(s)**	**Duration of Treatment**	**Findings**	**Ref**
Francisco et al. 2022	Wistar rats	300 mg/kg of body weight	63 days	Resveratrol reversed the male reproductive damage caused by nicotine.	[25]
Illiano et al. 2020	Human	150 mg/day	3 and 6 months	Multivitamin supplement based on resveratrol improves sperm motility and concentration.	[26]
Wang et al. 2024	Sprague-Dawley rats	5 and 20 mg/kg of body weight	6 weeks	Resveratrol induces a protective effect through autophagy and inflammation modulation.	[27]
Simas et al. 2021	Wistar rats	150 mg/kg of body weight	77 days	Resveratrol attenuated lipid peroxidation and sperm DNA damage.	[28]
Baazm et al. 2023	Wistar rats	20 and 50 mg/kg of body weight	60 days	Resveratrol reduces DNA damage in sperm cells, improving tissue health in the testes, and Increasing the production of proteins essential for proper sperm formation.	[29]
Singh et al. 2017	Albino mice	1 mg/kg of body weight	28 days	Role of resveratrol against cisplatin-induced testicular damage.	[30]
Hassanin et al. 2024	Albino rats	20 mg/kg of body weight	2 months	Administration of resveratrol protects against atrazine toxicity.	[31]
Özatik et al. 2017	Albino mice	320 mg/kg of body weight	28 days	Resveratrol cause improvement in testosterone, LH, FSH , the and apoptotic index.	[32]

RSV demonstrates significant phytoestrogen properties, effectively replicating certain estrogenic effects within the body. Estrogens, synthesized by Leydig cells in human testes, play a crucial paracrine regulatory role in male germ cells. This intrinsic estrogen production underscores the intricate balance between androgen and estrogen signaling pathways in male reproductive tissues [7][8]. Research indicates that RSV enhances hormone-induced estrogenic effects by functioning as a modulator of estrogen-response systems. Through activation of estrogen receptors, it exerts influence over various aspects of male reproductive function, encompassing spermatogenesis and testicular development. Beyond its estrogen-modulating effects, RSV engages in interactions with the androgen receptor. Specifically, it inhibits the dimerization of the androgen receptor, affecting transcription factors integral to testicular function. This interaction may modulate AR activity, potentially influencing diverse facets of male reproductive biology [9][10].

Additionally, RSV serves as a potent activator of sirtuin 1 (SIRT-1), an enzyme that plays pivotal roles in metabolism, energy production, and cell survival. The activation of SIRT-1 contributes to improved spermatogenesis through enhanced metabolic regulation within testicular cells and protection against oxidative stress and cellular damage in sperm cells [11]. RSV exhibits remarkable properties and plays a crucial role in activating adenosine monophosphate-activated protein kinase (AMPK), leading to numerous advantageous effects. This compound regulates sperm motility and maintains quality over extended storage durations, potentially enhancing fertility preservation techniques. RSV also enhances the fertilizing capacity of frozen mouse spermatozoa, offering promising insights into cryopreservation methods. Furthermore, RSV significantly contributes to the overall metabolic health of reproductive cells, suggesting potential applications in addressing fertility-related issues and optimizing reproductive outcomes [12][13].

4.1. RSV Improves Sperm Quality

RSV’s capacity to improve mitochondrial function and activity in sperm cells is crucial for sperm motility and quality. Mitochondria play a vital role in energy production for sperm movement, and any enhancement in mitochondrial function would likely translate to improved sperm motility and overall quality [14]. Recent scientific discoveries have revealed compelling evidence supporting the efficacy of RSV supplementation in enhancing male reproductive health. Clinical trials have demonstrated that patients receiving an RSV-based nutraceutical formulation, combined with vitamins exhibited significant increases in both sperm concentration and total sperm count [14]. Studies have also shown that RSV enhances motility and reduces DNA fragmentation in sperm cells. [15][16].

4.2.RSV Mitigates Oxidative Stress

RSV’s potent antioxidant properties play a pivotal role in combating oxidative stress, a primary factor contributing to male infertility. By activating anti-oxidant enzymes and neutralizing free radicals throughout the body, RSV significantly reduces oxidative damage, thus creating an environment conducive to optimal sperm development and function. Additionally, this compound enhances the overall antioxidant capacity of cells, protecting cellular structures from oxidative degradation, particularly in the testes where sperm production occurs [17].

The mitigation of lipid peroxidation, a form of oxidative damage that alters membrane fluidity and permeability, is another significant benefit of RSV. This reduction in lipid peroxidation leads to enhanced sperm motility and improved interaction between sperm and oocyte, ultimately contributing to improved fertility outcomes [14]. RSV may also help reduce protein modifications caused by oxidative stress. This reduction in protein modification can lead to improved ATP production in sperm cells, further enhancing their functionality and overall reproductive performance [18]. Lastly, RSV effectively activates antioxidant defense pathways, particularly through the Nrf2 pathway, significantly boosting cellular antioxidant defenses [19]. It also upregulates the expression of antioxidant proteins like heme oxygenase-1, contributing to its potential to combat oxidative stress and promote overall cellular health [20].

4.3.RSV Suppressed Inflammation

Inflammation plays a crucial role in male infertility. RSV, known for its potent anti-inflammatory properties, may offer promise in addressing this issue. This versatile compound can affect male fertility through multiple mechanisms. It inhibits crucial enzymes within the inflammatory cascade, notably cyclooxygenase (COX) and lipoxygenase (LOX), thereby disrupting the propagation of inflammatory signals [21]. RSV suppresses the production of pro-inflammatory cytokines, including IL-1α, IL-6, TNF-α, and IL-17, thus mitigating the body’s inflammatory response [22]. By modulating nuclear factor-kappa B (NF-κB) activation, RSV influences gene expression related to inflammation [23]. Additionally, RSV inhibits inducible nitric oxide synthase, further dampening inflammatory processes [24]. These multifaceted properties position RSV as a compound with significant therapeutic potential in preserving reproductive function and combating inflammation-driven fertility issues. Sudies on Resveratrol’s impact on male fertility are described in Table-1.

## 5. Nutritional Recommendations

Based on current knowledge, several nutritional recommendations can be formulated regarding RSV supplementation for male fertility. A pivotal clinical study employing 150 mg of RSV daily yielded encouraging outcomes. Notably, RSV is frequently co-administered with vitamins D, B6, B12, and folic acid. Research indicates progressive improvements spanning 1-6 months. It is crucial to acknowledge, however, that these recommendations are grounded in limited evidence and should be approached with caution [14]. When prescribing RSV supplementation, various factors must be meticulously considered. The efficacy may significantly differ depending on the underlying cause of infertility, emphasizing the importance of personalized treatment strategies. Potential synergistic interactions may arise when combining RSV with other fertility interventions, such as varicocele repair. Nevertheless, the long-term safety profile and potential adverse effects of RSV supplementation warrant further investigation. Moreover, the optimal dosage and duration of supplementation remain ambiguous, necessitating ongoing research to establish definitive guidelines. While the available evidence suggests promising potential for RSV in addressing male fertility issues, the current research foundation exhibits several limitations. Most studies have been conducted on a modest scale or in animal models, underscoring the imperative for larger human clinical trials. Additionally, the specific molecular pathways underlying RSV’s effects on male fertility require further elucidation. Perhaps most critically, optimal dosing regimens have not been conclusively established, highlighting the necessity for more comprehensive research in this domain. Future research should prioritize conducting larger, longer-duration human clinical trials to provide more robust evidence for the efficacy and safety of RSV supplementation. Elucidating the specific molecular mechanisms of RSV’s effects on male fertility would substantially enhance our comprehension of its therapeutic potential. Lastly, investigating optimal dosing regimens tailored to individual patient profiles could assist in maximizing the effectiveness of RSV supplementation while minimizing potential risks.

## Conclusion

The current review offered an exploration of the intricate connection between RSV and male fertility. Our investigation delved into the intricate causality of male infertility, emphasizing the pivotal role of oxidative stress and inflammation in undermining male reproductive well-being. RSV’s multifaceted attributes, encompassing its potent antioxidant, anti-inflammatory, and estrogen-regulatory properties, position it as a highly promising therapeutic agent in combating male infertility challenges.

## Conflict of Interest

The authors declare that there is no conflict of interest.
